# Integrating an aerosolized drug delivery device with conventional static cultures and a dynamic airway barrier microphysiological system

**DOI:** 10.1063/5.0100019

**Published:** 2022-09-13

**Authors:** Nikita Karra, Joao Fernandes, Emily Jane Swindle, Hywel Morgan

**Affiliations:** 1 Electronics and Computer Science, Faculty of Physical Sciences and Engineering, University of Southampton, United Kingdom; 2 Clinical and Experimental Sciences, Faculty of Medicine, University of Southampton, United Kingdom; 3 Institute for Life Sciences, University of Southampton, United Kingdom

## Abstract

Organ on a chip or microphysiological systems (MPSs) aim to resolve current challenges surrounding drug discovery and development resulting from an unrepresentative static cell culture or animal models that are traditionally used by generating a more physiologically relevant environment. Many different airway MPSs have been developed that mimic alveolar or bronchial interfaces, but few methods for aerosol drug delivery at the air–liquid interface exist. This work demonstrates a compact Surface Acoustic Wave (SAW) drug delivery device that generates an aerosol of respirable size for delivery of compounds directly onto polarized or differentiated epithelial cell cultures within an airway barrier MPS and conventional static inserts. As proof of principle, the SAW drug delivery device was used to nebulize viral dsRNA analog poly I:C and steroids fluticasone and dexamethasone without disrupting their biological function.

## INTRODUCTION

Current drug development and testing processes are both time consuming and expensive, with high failure rates (>90%) due to safety and efficacy concerns resulting from the use of simplistic static *in vitro* models or complex animal models subjected to species differences.^1–3^ These impediments have led to the generation of complex and dynamic *in vitro* models termed “organ on a chip” (OoC) or “microphysiological systems” (MPSs), which aim to recapitulate different organ functions, interfaces, and barriers within the human body. The airway epithelium is central to the maintenance of tissue homeostasis in the lung and acts as a physical, chemical, and immunological barrier.^4^ Models of the airways have been developed to replicate the bronchial barrier^5–10^ or alveolar regions^11–14^ of the lung, primarily using two branched^6^ or non-branched^5–17^ channels or chambers separated by a porous membrane that facilitates the culture of epithelial and/or endothelial cells on respective sides. Microfluidic systems enable the recapitulation of interstitial flow *in vivo*, while supplying cells with essential nutrients, removing waste and applying forces synonymous with the human microenvironment.^10^ Some models of the lung incorporate the real-time analysis of barrier integrity via impedance spectroscopy^5–19^ and mechanical stretch to mimic cyclical pressures in the alveoli.^11–13^

Airway microphysiological systems (MPSs) are a promising alternative to conventional models; however, methods need to be developed for delivery of compounds in a physiologically relevant manner, i.e., as an aerosol that better recapitulates human airway physiology with respect to respiratory diseases, as this can influence the deposition pattern and size preference of aerosols due to airway remodeling and constriction.^20^ Conventional methods of drug delivery such as inertial or electrostatic impactors and impingers cannot be easily miniaturized, and consequently, compounds are generally deposited in a liquid suspension.^7–21^ MPSs have been integrated with a collision-type atomizer^6^ or a mesh nebulizer^22–24^ with polydimethylsiloxane (PDMS) channels mimicking airway bifurcations^6^ or a large stagnant chamber (ALICE-Cloud) where drugs deposit via sedimentation onto an open-plate device.^23^ However, issues with these devices include the use of PDMS,^6^ which can leach or absorb compounds, excessive sample use due to non-specific deposition^23^ or to facilitate adequate dosing, sample solubility, viscosity, and degradation via shearing or unfolding in mesh nebulizers^22–24^ as highlighted recently.^25^ Thus, there is a necessity to develop a new compact airway barrier MPS compatible drug delivery device without the need for size sorting, with direct deposition of a correctly sized aerosol droplet containing compounds.

Surface acoustic wave (SAW) devices generate aerosols of respirable sizes (1–10 *μ*m) without meshes, blockages, frequent cleaning, or damage to sample integrity found with mesh nebulizers.^26–29^ SAW devices can nebulize monoclonal antibodies, plasmid DNA, yeast and mesenchymal stem cells without causing cavitation, degradation, or impacting cellular viability while suppressing large shear forces.^26–28^ It is considered a gentler process of nebulization,^26,27^ with dose controlled through nebulization time^28,29^ offering a potential drug delivery method that can be integrated with MPS technology.

**FIG. 1.**
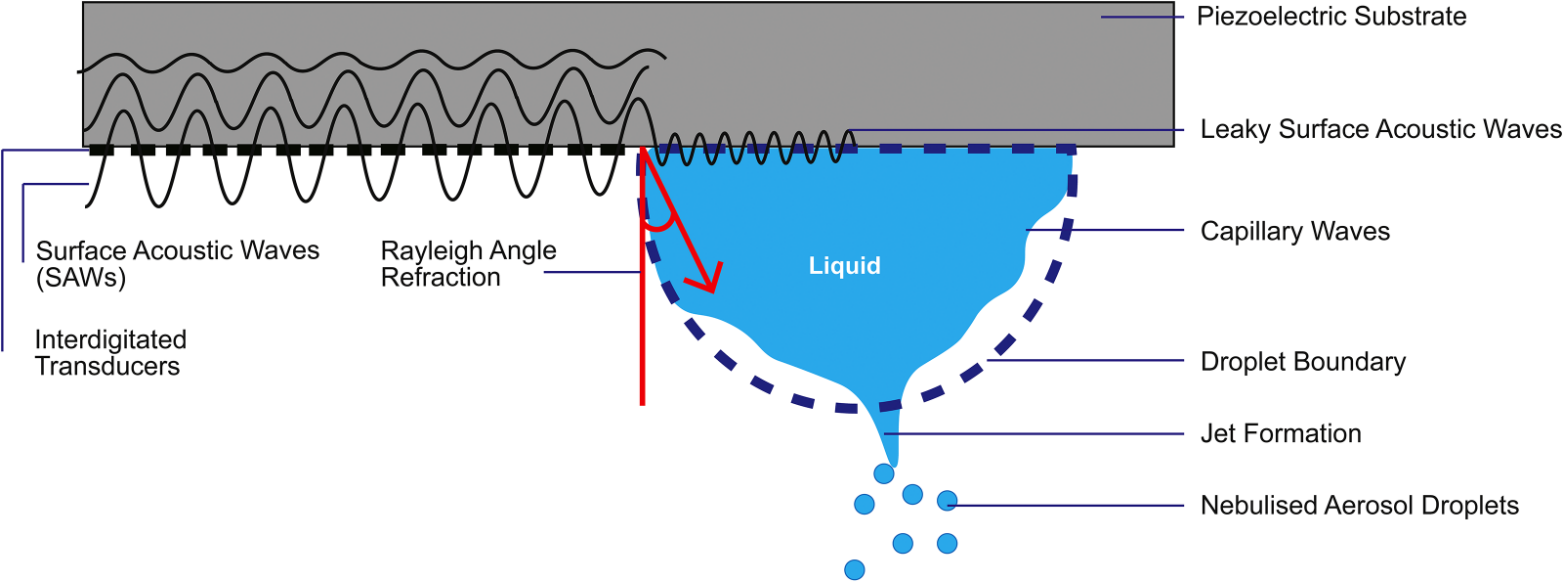
Surface acoustic wave nebulization. An AC voltage applied to a piezoelectric substrate causes relaxation and compression of the material generating surface acoustic waves that propagate transversely and longitudinally. If a high-frequency SAW reaches a liquid interface (e.g., droplet), the wave refracts at the Rayleigh angle, leading to destabilization of droplet boundary, jet formation, and nebulized aerosolized droplets. Image modified based on Ref. 34.

SAWs are generated by piezoelectric materials such a lithium niobate. The surface is patterned with an array of interdigitated electrodes and the application of a high-frequency AC voltage creates mechanical perturbation generating a surface travelling wave, with the electrode width and spacing determining the frequency. Upon interaction with a liquid interface (droplets or thin films), the wave destabilizes the liquid boundary through a refracted longitudinal pressure wave at the Rayleigh angle. This generates unstable capillary waves at the liquid boundary, leading to the formation of jets that break off to form aerosolized droplets of pre-determined size,^30–33^ as illustrated in Fig. 1.

This paper describes a simple and compact SAW device for direct aerosolized compound-delivery to polarized or differentiated human bronchial epithelial cell cultures grown in a microfluidic MPS at a liquid–liquid (LLI) or air–liquid (ALI) interface. Airway barrier formation and disruption were monitored in real-time using transepithelial electrical resistance (TER) measurements and confirmed by immunofluorescent staining for tight junctional proteins. Cells were challenged with aerosolized poly I:C, a dsRNA analog that mimics viral infection, and with basolateral tumour necrosis factor alpha (TNF-α) as a model of inflammation in the presence or absence of aerosolized steroids.

## METHODS AND MATERIALS

### Airway barrier MPS

The airway barrier MPS^5^ (see supplementary material 10) consists of eight individual microfluidic chips and manifolds, housed in a custom polymethylmethacylate (PMMA) stand. Media are perfused through the microfluidic chip with a syringe pump and commercial bubble traps (Darwin Microfluidics). The platform has easily exchangeable chips (held with magnets), connected to an impedance analyzer with plug-and-play functionality. The entire platform is controlled using a web-enabled interface. For further details, see Fernandes *et al*. (2022).^5^

Each microfluidic chip comprises a glass substrate onto which pairs of electrodes are patterned to record the TER of the cells. A 10 mm high PMMA structure forms the apical chamber, and the microfluidic channel is made from a 275 *μ*m thick laser cut tape and PMMA. The two halves are separated by a high porosity (12 *μ*m thickness, 0.4 *μ*m pore diameter, 1 × 10^8^ pores/cm^2^, PVP coating) polyester membrane (it4ip). The microfluidic manifold was milled out of polyetheretherketone (PEEK).

Prior to incorporation of cells, the system was cleaned with 1:50 bleach (<5% sodium hypochlorite), followed by copious rinsing with sterile de-ionized (DI) water.

### SAW device

**FIG. 2.**
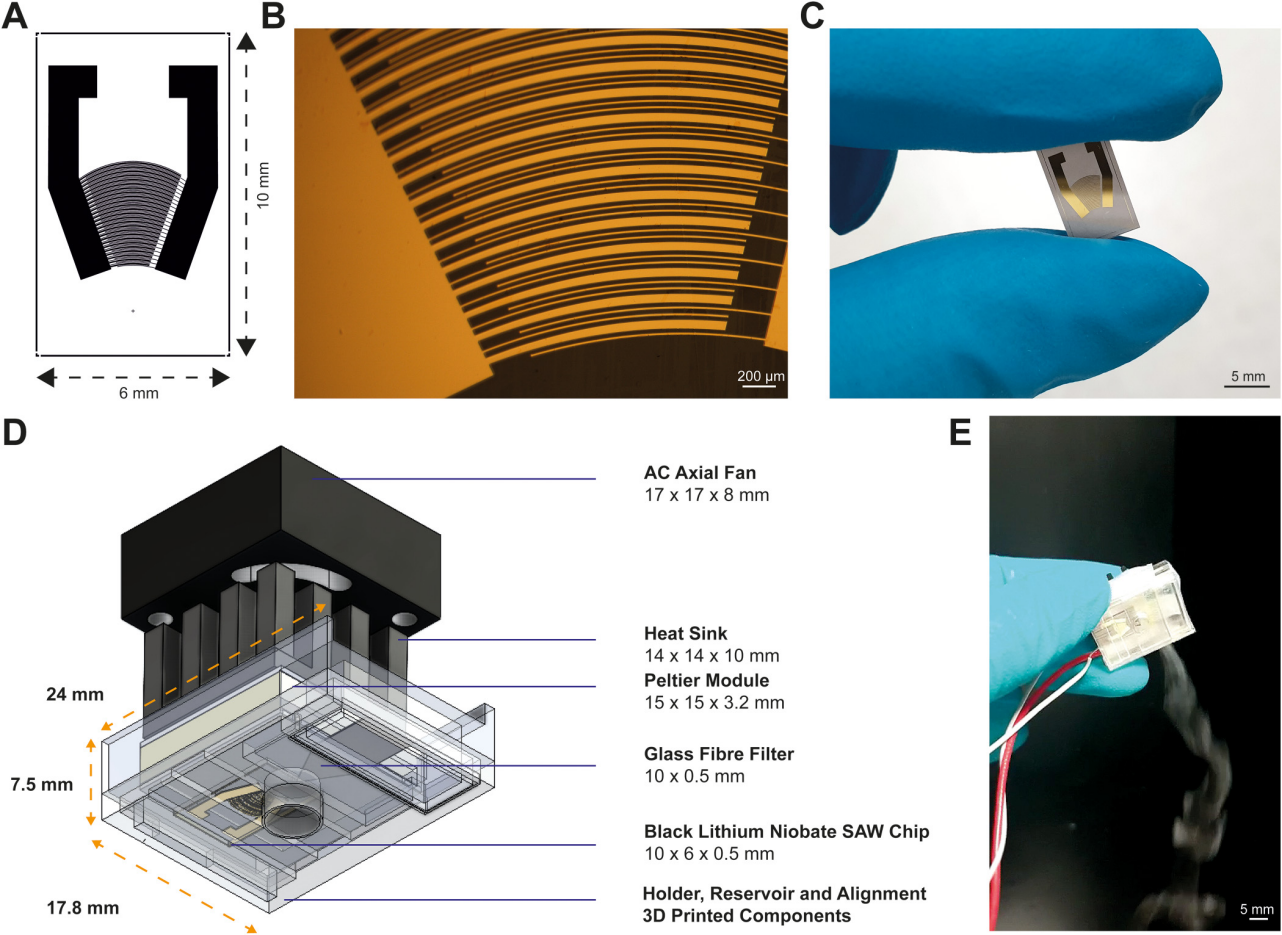
SAW drug delivery device overview. (a) Patterned lithium niobate chip. (b) Image of an interdigitated electrode array, consisting of 25 sets of electrode pairs on the negative port with widths of 17 and 49 *μ*m (−) and one electrode with a width of 17 *μ*m on the positive port (+) with 17 *μ*m spacing, generating a wavelength of 130 *μ*m (5× magnification). (c) Image of the fabricated SAW chip. (d) Delivery device showing a SAW chip, a glass fiber sample reservoir, a temperature regulator, and a 3D printed holder. (e) Image of aerosol with the device actuated using a modulated signal with varying amplitudes from 28 to 40 V_p-p_ (lowest to highest) when using an operating frequency of 30.62 MHz and a modulation frequency of 1 kHz.

The SAW drug delivery device comprises a patterned black lithium niobate chip, a Peltier cooler, a fluid delivery mechanism, and a custom 3D holder (Fig. 2). The chip is patterned with curved interdigitated electrodes following the single-phase unidirectional transducer (SPUDT) design of Shilton *et al*. and Qi *et al*.^35,36^ to enable greater focusing of acoustic energy.^35^ The device has 25 pairs of electrodes with a width of 17 or 49 *μ*m and a spacing of 17 *μ*m, generating an oscillation with a wavelength of 132 *μ*m and an operating frequency of 30.62 MHz [see Figs. 2(a)–2(c)]. Electrode arrays were fabricated on a Y-cut, X propagating 128° black lithium niobate wafer (Roditi) from a layer of 10 nm chromium and 60 nm gold patterned by standard photolithography. The wafer was diced into individual chips, which were then soldered to a ribbon cable and bonded to a Peltier cooler with heat sink using a pressure-sensitive tape and mounted in a custom 3D-printed holder with the glass fiber filter.

A Peltier cooler and fan [Fig. 2(d)] maintained the chip surface at ∼38 °C (see supplementary material 1). The device was driven with voltages ranging from 28 to 40 V_p-p_ at 30.62 MHz, with modulation at 1 kHz to reduce the total power and increase nebulization efficiency.^37^ The temperature remained constant during 5 min of continuous actuation. A continuous supply of liquid was provided to the chip from a glass fiber filter, and the nebulization rate was ∼20 *μ*l/min via Schlichting streaming^32^ and Eckart streaming.^32–38^

**FIG. 3.**
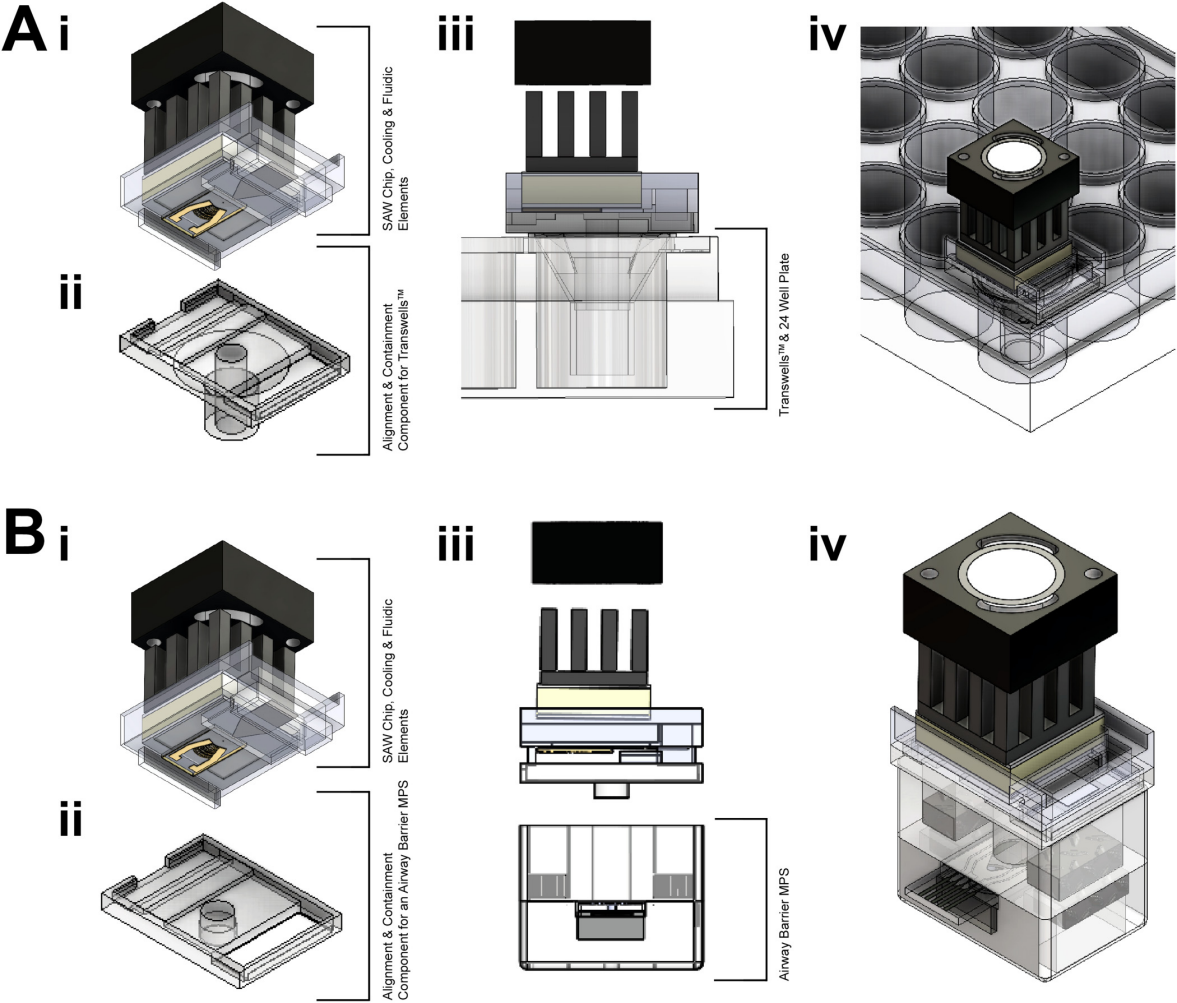
(a) Overview of the SAW device with a patterned lithium niobate substrate, active cooling mechanisms (heat sink and Peltier element), a fluid reservoir (a glass fiber filter), and a 3D printed holder [a(i) and b(i)], in addition to the alignment and containment pieces for Transwells™ [a(ii)] and the airway barrier MPS, respectively [b(ii)]. Schematic showing the device paced above conventional Transwells™ [a(iii) and (a)(iv)] and the airway barrier MPS [b(iii) and b(iv)].

The SAW chip was mounted on commercial nanoporous membrane inserts (Transwell™ supports) and also on the airway barrier MPS previously described in Fernandes *et al*.^5^ using custom 3D printed holders so that aerosols were directly introduced into the apical chamber (Fig. 3). The complete SAW device (Fig. 2) was connected a high-power amplifier (Mini Circuits, ZHL-5W-1+) and a signal generator (Tektronix TDS2014C). Prior to use, the SAW device was decontaminated with 70% ethanol and assembled in a MSCII.

### Cell culture

Two human bronchial epithelial cell lines were used: the 16HBE14o- cell line and the basal cell immortalized non-smoker cell line BSi-NS1.1, chosen for their ability to form a polarized^39^ or polarized and differentiated epithelial barrier^40^ on nanoporous membranes, respectively. The 16HBE14o- cell line was maintained in a minimal essential medium (MEM) with Glutamax (Gibco) supplemented with 1% penicillin–streptomycin and 10% foetal bovine serum (FBS) (Life Technologies). The BCi-NS1.1 cell line was maintained in Pneumacult™ Ex Plus Complete media [PneumaCult™-EX Plus Medium supplemented with PneumaCult™-Ex Plus supplement (1×), 1% penicillin–streptomycin, 20 U/ml nystatin and 0.096 *μ*g/ml hydrocortisone] solution.

Cells were seeded at densities of 7.6 × 10^5^ cells/cm^2^ (16HBE14o- cells) or 4.5 × 10^5^ cells/cm^2^ (BCi-NS1.1 cells) onto collagen coated [30 *μ*g/ml collagen (Advanced Biomatrix)] 0.4 *μ*m PET membranes (12 *μ*m, 0.4 *μ*m pore diameter, 1 × 10^8^ pores/cm^2^, PVP coated) in the microfluidic chip, or 0.4 *μ*m PET membranes (10 *μ*m, 0.4 *μ*m pore diameter, 4 × 10^6^ pores/cm^2^, cell culture treated) in Transwell™ inserts (Corning) for 1 h to facilitate cell adhesion (without flow). After cell adhesion, fluid was pumped through the chips (30 *μ*l/h) and the electrical impedance was measured every 17 min. For Transwells™, TER measurements were performed using chopstick electrodes and an ERS-2™ Millicell Voltohmmeter (MERS00002, Merck). Data were recorded daily for 16HBE14o- cells and weekly for BCi-NS1.1 cells [after a 15-min incubation at 37 °C with Hanks buffered saline solution (100 *μ*l)]. Apical and basolateral media were replaced on days 2 and 4 for 16HBE14o- cells while basolateral media were replaced 3× weekly for BCi.NS1.1 cells.

### Air–liquid interface cell culture

For optimal growth and polarization, the 16HBE14o- cells prefer a liquid–liquid interface.^41^ In order to challenge cells at ALI, after barrier formation (>300 Ω cm^2^) on day 5, the apical volume was removed, and cells were incubated at ALI for between 0 and 4 h to determine the optimal time. The TER was measured by submerging the cells in equilibrated media and taking readings hourly thereafter up to 5 h and then again at 24 h. BCi-NS1.1 cells were taken to ALI after 2–3 days of submersion in Pneumacult™ Ex Plus Complete media by removing the apical media and replacing the basolateral media with Pneumacult™ ALI Maintenance media [PneumaCult™-ALI base medium supplemented with PneumaCult™ ALI supplement (1×), PneumaCult™ ALI maintenance supplement (1×), 1% penicillin–streptomycin, 20 U/ml nystatin, 4 *μ*g/ml heparin solution, and 0.48 *μ*g/ml hydrocortisone solution] and cultured for four weeks with basolateral media replaced 3× weekly.

### dsRNA analog or tumour necrosis factor-α challenge with steroid treatment

Cells were challenged on day 5 (16HBE14o-) or day 28 (BCi-NS1.1) after formation of an epithelial barrier (>300 Ω cm^2^). All aerosolized challenges were performed in an MSCII and the deposition amount controlled by the nebulization rate (20 *μ*l/min). The cells are temperature sensitive,^42^ so only one or two chips/culture were removed at a time.

### Liquid-liquid interface

The 16HBE14o- cells were challenged by removing 20 *μ*l of the medium from the apical compartment and replacing it with 20 *μ*l of poly I:C (25 *μ*g/ml, a dsRNA analog as a mimic of viral replication), fluticasone propionate (100 nM) or poly I:C, and fluticasone propionate together or media alone as a control. In each case, samples were either pipetted or nebulized (onto the bulk liquid).

### Air–liquid interface

For 16HBE14o- cells, the medium was replaced the day before challenge. Challenge required removal of the entire apical volume, followed by addition of 20 *μ*l poly I:C (either nebulized or pipetted directly onto cells) followed by the addition of 180 *μ*l (static Transwell™ cultures) or 80 *μ*l (airway barrier MPS) equilibrated media without compounds to provide a final poly I:C concentration of 25 *μ*g/ml. Media alone were used as a control.

For BCi-NS1.1 cells, the basolateral medium was replaced with Pneumacult™ ALI Maintenance media without hydrocortisone the day before challenge. The following day, 20 *μ*l of dexamethasone (100 nM) was either nebulized or pipetted into the apical compartment, with or without TNF-α (1 ng/ml) pipetted in the basolateral compartment. Controls consisted of apically pipetted starvation media (Pneumacult™ ALI Maintenance media without hydrocortisone) or nebulized starvation media with TNF-α.

### Laser diffraction

The size of aerosol droplets produced by the SAW device was measured using the Malvern Spraytech system STP5315. The Spraytech system measured for 1 min of continuous nebulization following initial background measurements, in which laser obscuration over 5% was recorded. The SAW device was placed 10 mm below the center of the laser and the device was actuated at 30.62 MHz with a modulation frequency of 1 kHz and 20% depth at voltages of 28/40 V_p-p_ (min/max). Triplicate measurements were recorded per each chip (three chips), where the values 30 s after signal actuation were averaged. Generation of a sustained plume was achieved through the use of a glass fiber filter (5 × 30 mm strip) positioned at the end of the chip, enabling a meniscus to be drawn out and nebulized when prewetted with 200 *μ*l DI water, from which the excess was removed and replaced with 20 *μ*l DI water, prior to actuation.

### Immunofluorescence staining and imaging

At the end of a challenge, cells were fixed in 4% PFA solution (Merck), washed, and then stored in 1× phosphate-buffered saline (PBS) at 4 °C. Cells were permeabilized (0.1% Triton X-100 in PBS, 30 min at room temperature) followed by blocking (PBS with 2% bovine serum albumin (BSA) and 0.1% Tween 20, 60 min at room temperature) before overnight incubation with Acti-stain555-phalloidin (Cytoskeleton, PHDH1-A) and AlexaFluor®488-conjugated mouse anti-human occludin antibody (Life Technologies, Clone OC-3F10) at 4 °C in a humidified chamber. Samples were then washed 3× (PBS with 0.1% Tween-20), counterstained with 4′,6-diamidino-2-phenylindole (DAPI) nuclear stain (Merck, 10236276001) for 15 min, and washed 3× (PBS with 0.1% Tween-20 solution) and 1× (dH_2_O) before mounting onto coverslips with Mowiol (Merck, 81381). Images were captured at 63× using confocal microscopy in the xyz mode (a Leica TCS-SP8 laser scanning microscope) with excitation wavelengths of 405 nm (DAPI), 561 nm (Actin), and 488 nm (Occludin). A Z-projection stack created with the Leica application suite using sequential scans to limit spectral bleeding.

### Data analysis

Results are presented as mean ± standard deviation (SD). Normality was assessed using a Shapiro–Wilk test. For 16HBE14o- cells at LLI, paired *t*-tests were used to compare day 0 and day 5 TER values and delivery methods (pipetted vs nebulized) while a one-way analysis of variance (ANOVA) was used to compare challenge conditions within delivery methods (Media, poly I:C, fluticasone, and combination). For the 16HBE14o- cell pipetted and nebulized at ALI, a Mann Whitney test was used to compare between conditions. For the challenge within the airway barrier-on-a-chip platform at ALI, an unpaired *t*-test was used. For the BCi-NS1.1 challenge, comparison between pipetted conditions used a paired *t*-test, while comparisons between pipetted and nebulized or within nebulized conditions used an unpaired *t*-test. Results were considered statistically significant when *p *≤ 0.05.

## RESULTS AND DISCUSSION

### Device characterization

**FIG. 4.**
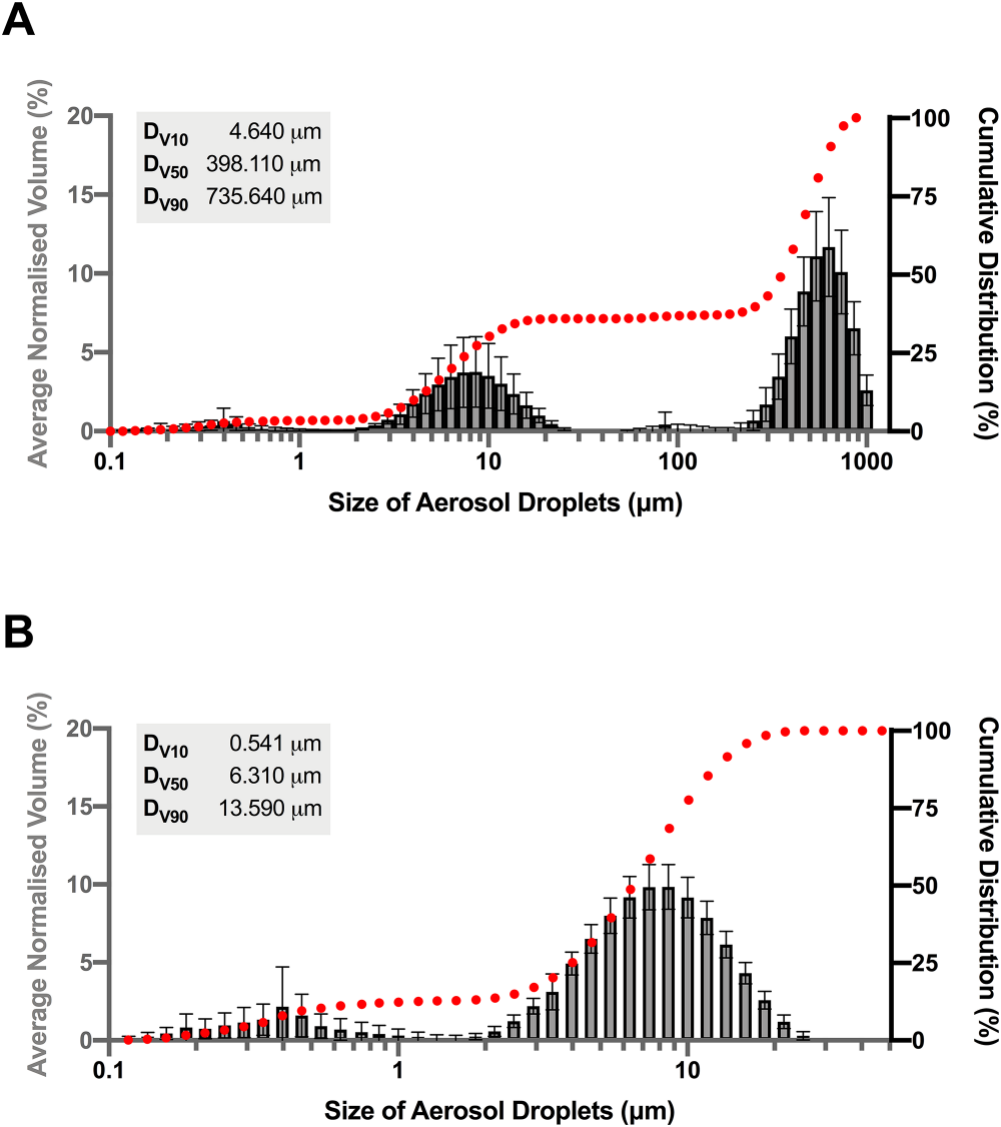
Aerosol droplet size distribution. The average droplet size distribution from the SAW device. Results show the entire normalized distribution (a) and the distribution after omitting large satellite droplets (b). The data are average and standard deviation of n = 3 devices in triplicate.

The aerosol size was determined by laser diffraction (Malvern Spraytech STP5315) and the results showed a trimodal distribution (Fig. 4), with peaks at 0.398, 8.58, and 630.96 *μ*m. The high incidence of large satellite droplets was a consequence of surplus water being drawn from the reservoir as a prototype fluid supply was used. The liquid bulk is thought to be deformed into a conical shape by Eckart streaming leading to the ejection of large droplets (following whipping and breakage) until an equilibrium state is achieved between the sample flow and nebulization rate.^32–44^ This phenomenon was captured in nebulization images (see supplementary material 2); at the start of actuation, multiple large droplets are visible, and these persist for 20 s after which they disappear leaving only the nebulized aerosol, giving a skew in aerosol distribution.

The scatter data were normalized to omit satellite droplets, and the final aerosol data are shown in Fig. 4. The D_V10_, D_V50_, and D_V90_ values are highlighted, indicating the percentage of droplets below that volume. In other words, 10% of the aerosol droplets fall below 0.514 *μ*m, 50% below 6.310 *μ*m, and 90% below 13.590 *μ*m, indicating that the aerosol is suitable for physiologically relevant direct deposition.^45^

### Modeling viral infection and treatment with the SAW device

The 16HBE14o- cell line was cultured on Transwell™ supports at a LLI for 5 days until the formation of an epithelial barrier (TER of 598 ± 204 Ω cm^2^, n = 3). To determine whether nebulization impaired the biological activity of compounds, poly I: C and a steroid were nebulized onto polarized cells, with media as a control. Poly I:C is used as a model of viral infection. It is a synthetic analog of dsRNA, which is used to mimic the dsRNA generated during replication of viruses in cells. Through activation of TLR3, poly I:C induces an inflammatory response and disassembly of tight junctional complexes in epithelial cells.^46^ Fluticasone is used to treat airway inflammation in asthmatic patients and exerts its effect on the epithelium by reducing inflammatory cytokine release (e.g., IL-8 and TNF-α)^47,48^ and enhancing airway function^48,49^ while also counteracting the poly I:C-induced disruption of barrier integrity.^47^

**FIG. 5.**
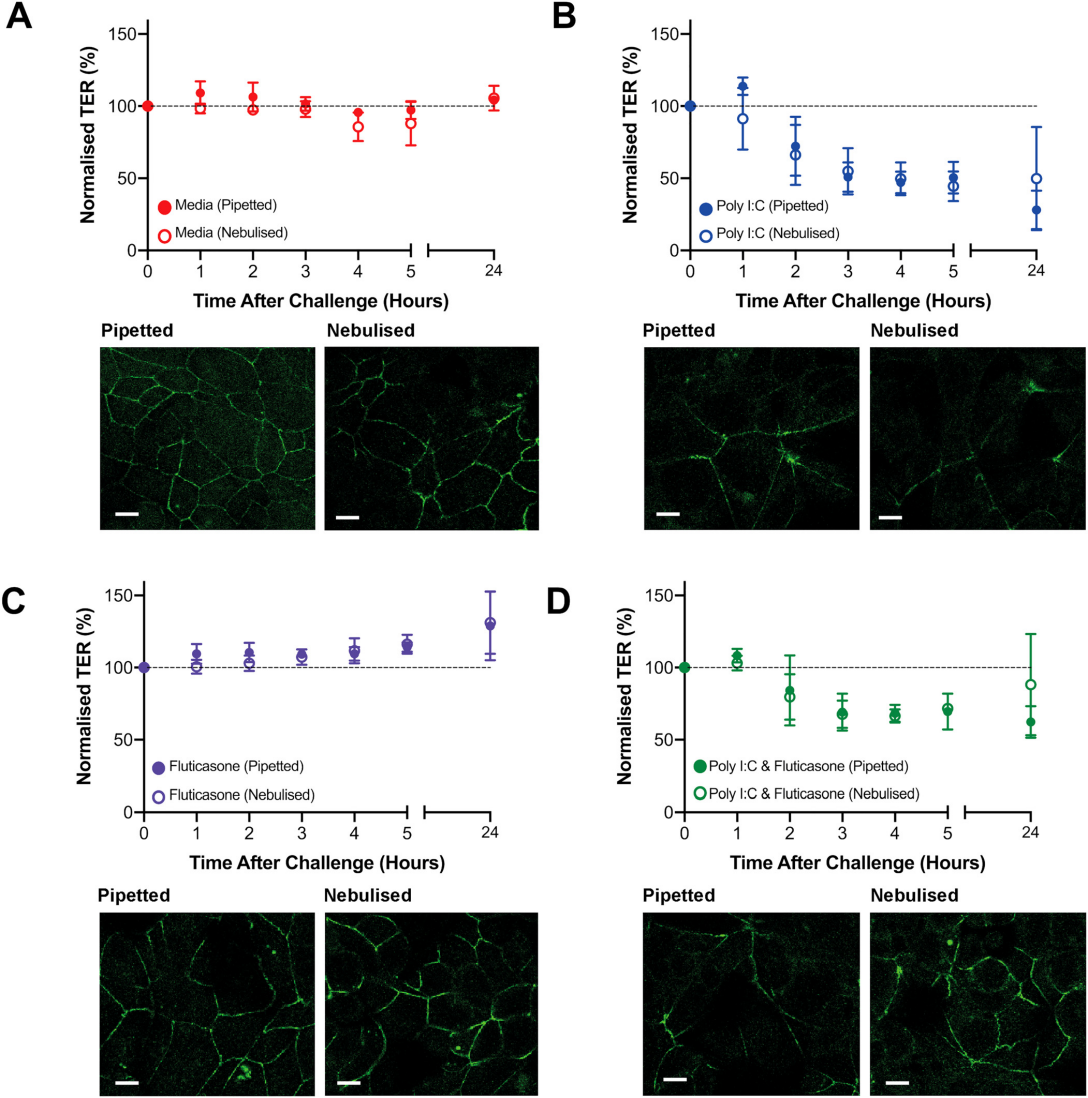
Barrier integrity following challenge with poly I:C and fluticasone. 16HBE14o-14o- cells were challenged at day 5 using either pipetted or nebulized stimulants. Cells were apically stimulated on day 5 with media only (a), 25 *μ*g/ml poly I:C (b), 100 nM fluticasone (c), or a combination of poly I:C and fluticasone (d) using pipetted or nebulized solutions at the LLI. The data are normalized to the day 5 value (set to 100%). Representative images show distribution of the tight junctional protein occludin (green). Images were captured using a Z projection stack at 63×, with a wavelength of 488 nm occludin (a Leica TCS laser scanning microscope). The white scale bar indicates 10 *μ*m. Results are means ± SD or representative immunofluorescent images, n = 3 biological repeats in duplicate for 1 SAW device.

To enable comparison between different conditions, the TER data (Fig. 5) were normalized to the initial value (i.e., day 5 = 100%). For media control, the TER remained stable over 24 h for both pipetted and nebulized samples [Fig. 5(a)] while a steep decrease in TER was observed between 0 and 3 h, (from 100% to around 50%) following poly I:C stimulation with no difference between pipetted and nebulized conditions [Fig. 5(b)]. When fluticasone was added to the cells, a significant increase in TER was observed at 5 h [(*p* = 0.03 pipetted) and *p* = 0.04 (nebulized)] but not at 24 h for both the pipetted and nebulized challenge [Fig. 5(c)]. When poly I:C and fluticasone were added simultaneously, a steep decrease in the first 3 h was observed (for both pipetted and nebulized conditions) [Fig. 5(d)] but with a smaller decrease than for poly I:C alone [Fig. 5(b)].

Irrespective of treatment, there was no significant difference between the effect of pipetted or nebulized delivery of poly I:C (*p* = 0.3), fluticasone (*p* = 0.63), or poly I:C and fluticasone combined (*p* = 0.21), indicating that these compounds could be nebulized without impacting their biological function. Irrespective of the delivery method (pipetted or nebulized), poly I:C induced a significant reduction in TER at 24h compared to the pipetted (*p *= 0.0003) and nebulized (*p *= 0.005) media control conditions (see supplementary material 3), which is similar to previous findings.^46–50^

In order to determine whether the TER measurements reflected the organization and distribution of epithelial cell-to-cell tight junctions, cells were immunofluorescently stained with an occludin-specific antibody (green) and counterstained for the actin cytoskeleton (red) and nuclei (DAPI, blue). For both the pipetted and nebulized media controls, a continuous apicolateral localization of occludin was observed clearly showing each intercellular border [Fig. 5(a)] with a well-organized actin cytoskeleton encircling each cell (see supplementary material 4). Following challenge with poly I:C, occludin was re-organized and showed loss of continuity at cell–cell junctions for both pipetted and nebulized conditions [Fig. 5(b)] and the actin cytoskeleton was more disordered (see supplementary material 4), with redistribution of actin bundles (multiple actin filaments) at pericellular junctions similar to previous studies.^10–46^ For cells treated with fluticasone alone, a similar pattern was observed as for media control [Fig. 5(c)] while for cells challenged with poly I:C and fluticasone [Fig. 5(d)], the pipetted samples displayed similar patterns to poly I:C, with localized occludin staining and disorganized actin, but the nebulized sample staining indicated some recovery as occludin staining started to become more continuous. Occludin only staining is shown in supplementary material 5 and represents a wider field of view. This is in line with the TER data where the nebulized combination had a higher TER than the pipetted samples (see supplementary material 3).

### Nebulizing poly I:C at the air–liquid interface

The 16HBE14o- cell line polarizes with differential apical-basolateral expression of tight junction proteins.^42–51^ These cells are preferentially maintained at a LLI to produce confluent tight barriers,^41,42^ but this is not representative of human airways where the epithelium is at an ALI. Consequently, to challenge cells in a more physiologically relevant manner (at ALI) without impacting the barrier integrity, the apical media were first removed and then immediately replaced after administration of a nebulized challenge (see supplementary material 6).

**FIG. 6.**
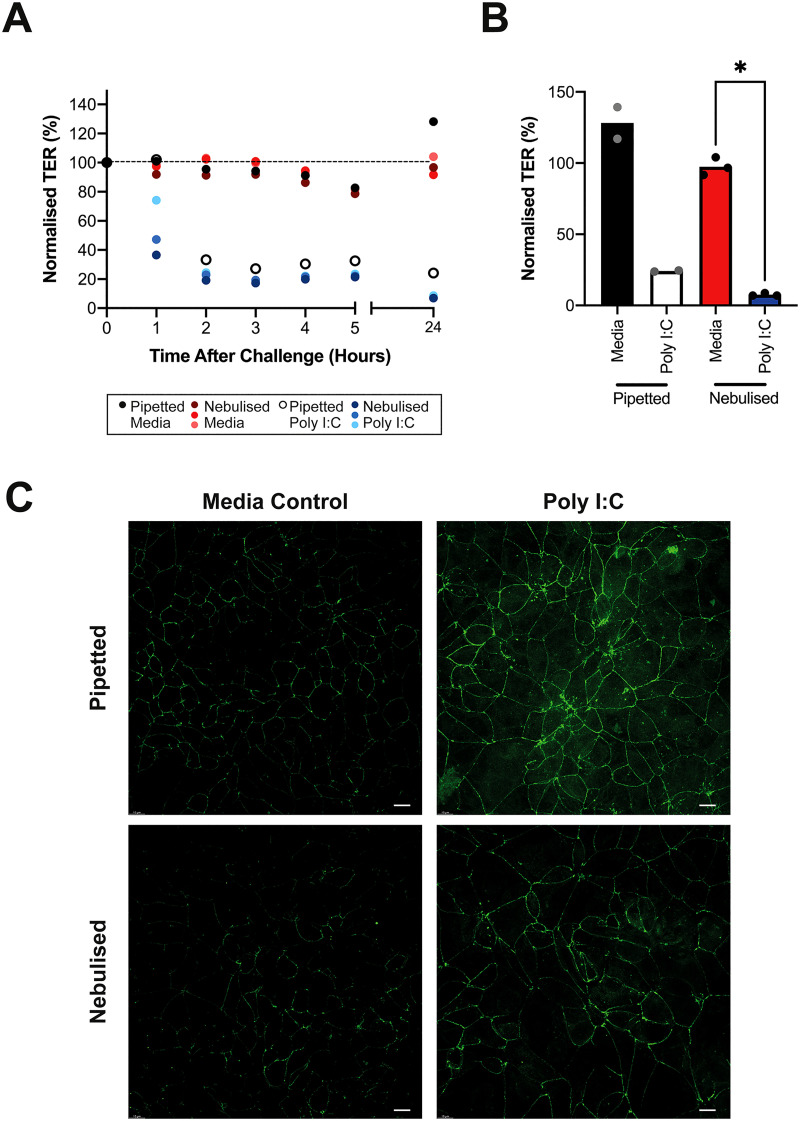
Nebulization at ALI using different SAW devices. TER data in (a) and (b) are normalized to the day 5 value (100%). Results are for n = 3 devices and one biological repeat in duplicate. Statistical analysis via Mann–Whitney test such that the * refers to *p* < 0.05 (c). Samples (n = 1 biological repeat in singlet) were immunofluorescently stained using DAPI nuclear (blue), actin cytoskeleton (red), and occludin tight junction (green). Images representative of n = 3 devices and the apicolateral region of the Z projection stack. The white scale bar indicates 10 *μ*m.

Compared to media controls, cells challenged with poly I:C showed a steep decrease in TER during the first 2 h, which was maintained up to 24 h [Figs. 6(a) and 6(b)]. A similar pattern of poly I:C-induced reduction in TER was observed when poly I:C was nebulized in three separate SAW devices. The TER results were confirmed by immunofluorescent staining of tight junctions, where media controls showed continuous pericellular localization of occludin with regular actin cytoskeletal organization, while following poly I:C stimulation, there was a discontinuous pericellular organization of occludin [Fig. 6(c) and see supplementary material 7]. These data demonstrate that compounds can be delivered by aerosol with good reproducibility between different devices (n = 3).

### Modeling bronchial TNF-α inflammation and steroidal recovery

To better emulate the bronchial epithelium *in vivo*, the BCi cell line was used at an ALI owing to its multipotent differentiation capacity.^40^ TNF-α is a proinflammatory cytokine abundantly found in asthmatic patients, which mediates the loss of barrier function via disruption or loss of tight junctional complexes.^52^ The glucocorticoid dexamethasone was investigated to evaluate its effectiveness in improving airway barrier function in the presence of TNF-α as it is a common therapeutic employed to treat asthmatic patients^53^ due to its anti-inflammatory properties^54^ and has been shown to inhibit TNFα-mediated tight junction disruption in a skin-on-a-chip model.^55^

**FIG. 7.**
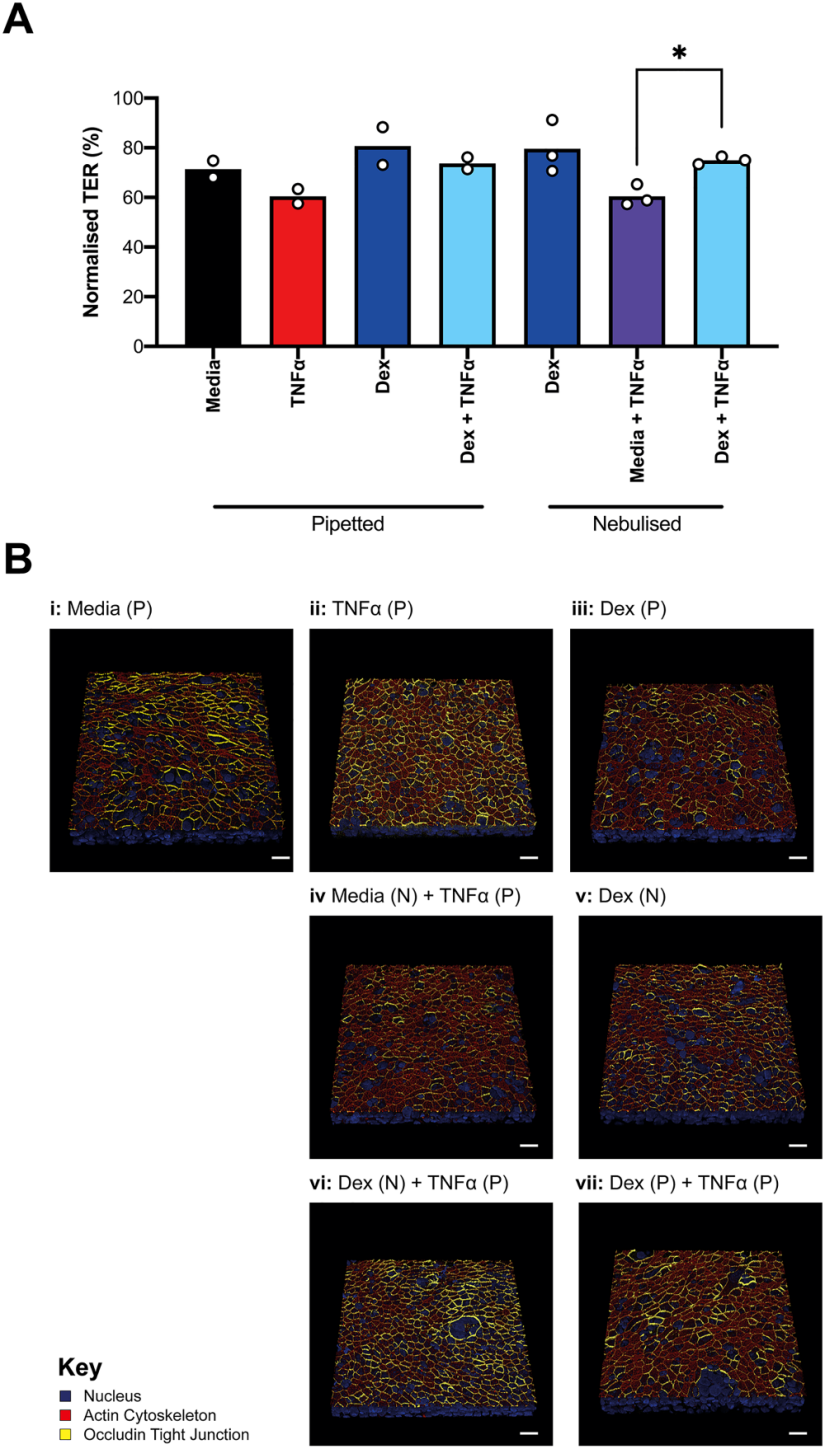
TER and immunofluorescent staining of BCi cells after TNF-α and dexamethasone exposure. BCi-NS1.1 cells were grown and differentiated for four weeks before challenge, and TER measured weekly (a). The TER data in (b) are normalized to week 4 value before challenge (100%) for n = 3 nebulization devices or 2 biological repeats for pipetted conditions, each biological repeat is represented by a white dot. Statistical analysis was performed via a paired t-test, such that * refers to *p* < 0.05 (c). Immunofluorescently stained representative samples (n = 3 devices) showing overlaid occludin tight junction (yellow), actin cytoskeleton (red), and nuclear (blue) staining. The white scale bar indicates 20 *μ*m.

BCi cells were basolaterally challenged with TNF-α (10 ng/ml) in the presence or the absence of apical treatment with pipetted or nebulized dexamethasone (100 nM) using only media or dexamethasone controls at ALI. The TER measurements at 24 h post-challenge [Fig. 7(a)] were normalized to the week 4 time-point before the addition of stimulants (see supplementary material 8).

The data [Fig. 7(a)] showed a decrease in TER for the media control (from 100% to 71% of baseline after 24 h), thought to occur due to the change to starvation media without hydrocortisone. Cells basolaterally stimulated with TNF-α showed the greatest reduction in TER, to 60% of baseline. This was counteracted by the apical addition of dexamethasone, generating a higher barrier integrity (74% and 75%) for pipetted and nebulized methods, respectively. For dexamethasone only, the TER was higher than the media control for both pipetted and nebulized conditions (81% and 80%), and there was little difference between TNF-α and dexamethasone-stimulated cells and dexamethasone alone, indicating barrier recovery. To confirm that this improved barrier function was due to dexamethasone and not a component in the media itself, media were apically nebulized with TNF-α pipetted basolaterally and showed similar TER values as TNF-α alone.

These results were corroborated by immunofluorescent staining, which analyzed the expression of differentiation markers (β-tubulin—ciliated cells, MUC5-AC—goblet cells: see supplementary material 9) in addition to tight junction formation (occludin) and actin cytoskeleton organization. As shown in Fig. 7(b), occludin is present in the apicolateral regions of the cell layer and is more pronounced in the presence of dexamethasone {Figs. 7[b(iii)], 7[b(v)], 7[b(vi)], and 7[b(vii)]} compared to the control of media alone. For TNF-α {Fig. 7[b(ii)]} or TNF-α and nebulized media {Fig. 7[b(iv)]}, the occludin organization is less prominent. The actin cytoskeleton organization is similar for all conditions. For TNF-α treated samples, the addition of dexamethasone shows increased intensity and organization of occludin at cell-to-cell junctions {Figs. 7[b(vi)] and 7[b(vii)]}. These results are similar to the literature, where dexamethasone has been shown to improve TER in bronchial epithelial samples challenged with TNF-α by inhibiting tight junction loss and disruption.^52^ These data demonstrate the potential use of aerosolization devices such as the presented method in better disease modeling and treatment during drug discovery and testing in the future.

### Integrating aerosolized drug delivery with an airway MPS

**FIG. 8.**
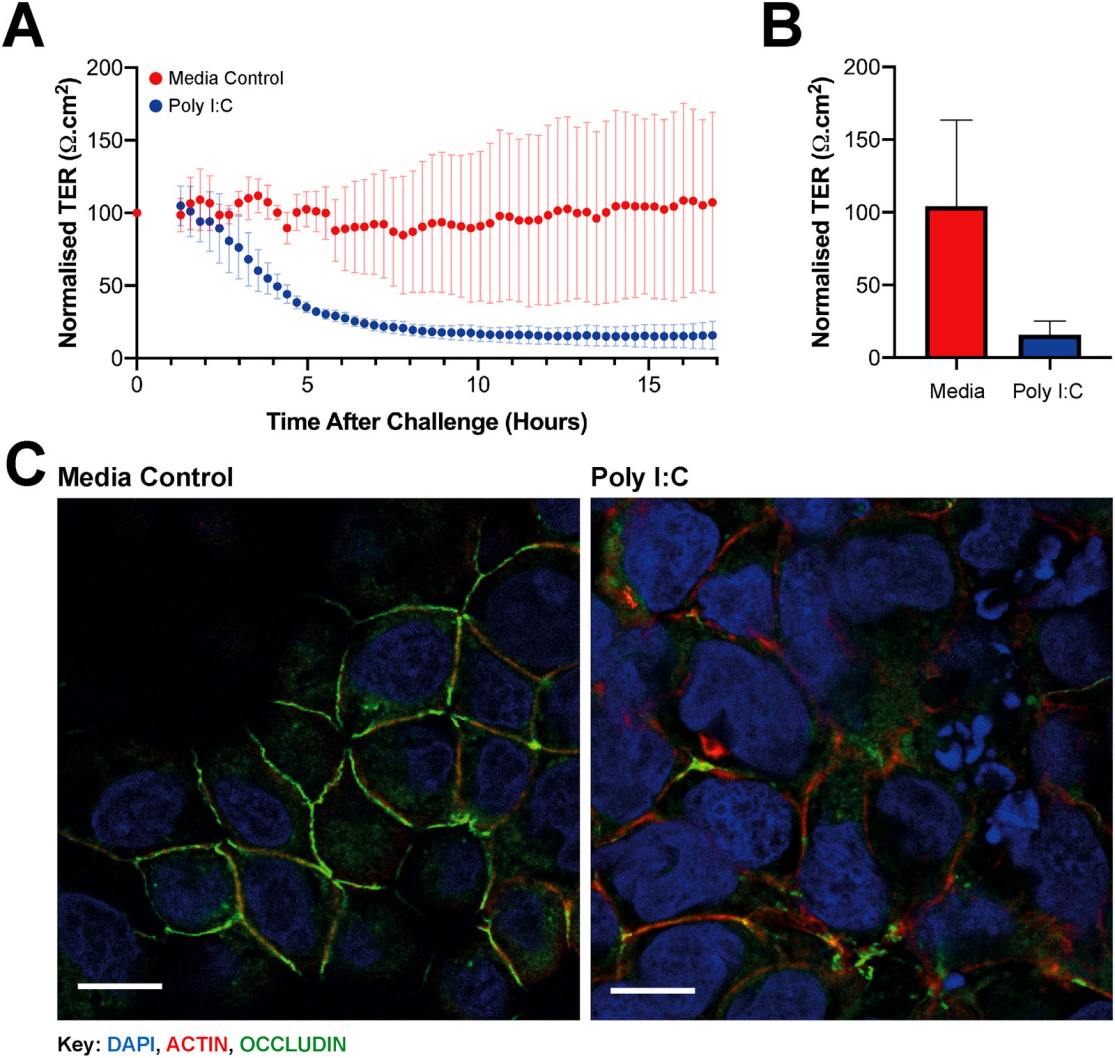
TER and immunofluorescent staining of cells grown in the microfluidic system. TER data (a) were normalized to the day 5 value (100%); the 24-h time point is in (b). Samples were fixed 16 h after challenge with 4% PFA and were immunofluorescently stained using DAPI nuclear (blue), actin cytoskeleton (red), and occludin tight junction staining (green). (c) Shows overlaid data with the white scale bar indicating 10 *μ*m. Data are for n = 3 devices; (a) and (b) show means ± SD and (c) immunofluorescent images.

The SAW device was used in conjunction with an airway barrier MPS detailed in Fernandes *et al*.,^5^ which incorporates micro-electrodes to measure TER by electrical impedance. The 16HBE14o- cell line was grown on-chip for five days until an epithelial barrier was established (see supplementary material 11). Poly I:C (25 *μ*g/ml) was then nebulized onto polarized bronchial epithelial cells, and barrier integrity was monitored in real time by TER measurements. Figure 8(a) shows the data normalized to the timepoint before challenge. These data show that the media control remains at around 100% over the course of nebulization, while poly I:C induced a decline to 16% of the pre-challenge value [Fig. 8(b)]. Immunostaining of samples showed the characteristic reorganization of occludin and actin organization [Fig. 8(c)], confirming that the immunofluorescent staining confirmed that the poly I:C treatment caused disruption of tight junctional complexes.

The variance in the TER data for the microfluidic platform is predominantly due to chip-to-chip variation because these are all manually fabricated which can cause discrepancies in the modelled data owing to variability in dimensions.^5^ The variability in SAW device-to-device deposition may be another factor, as compound loss can occur onto holders because vortexes are formed in the chamber as the aerosol is nebulized before sedimenting onto the cells, similar to the ALICE cloud system^56^ but on a much smaller scale. The SAW system uses much smaller sample volumes (20–40 *μ*l) compared with 0.5–5 ml for the ALICE cloud platform.^57^ The significantly smaller footprint of the SAW nebulizer means that multiple systems can be used together for delivery of different compounds in an enclosed environment, limiting crossover and contamination, which is advantageous when compared with the ALICE cloud platform that nebulizes non-specifically over the entire device and surrounding area. The ALICE cloud also employs mesh nebulizers that can lead to sample damage via unfolding or shearing are difficult to clean and can be blocked.^57–60^ Highlighting the potential for a SAW-based drug delivery platform to directly deposit compounds without compromising their biological function.

## CONCLUSIONS

A compact drug delivery device has been developed to provide a simple means of delivering aerosols directly onto cells grown in both conventional static (Transwell™) and airway barrier MPS. The SAW device generates a fine aerosol plume with 90% of droplets of respirable size (<13.59 *μ*m) at a nebulization rate of 20 *μ*l/min, while maintaining a surface temperature of ∼38 °C. The device was integrated with cell culture chambers using 3D-printed holders enabling direct deposition onto cells while minimizing loss to the external environment. The device was used to deposit compounds such as poly I:C and steroids (fluticasone and dexamethasone) directly onto cells at air–liquid and liquid–liquid interfaces without loss of biological activity when compared to controls. The device provides a simple way of delivering compounds to airway barrier MPS for future drug development and testing and will help to develop more relevant models with aerosol drug delivery at an ALI.

## SUPPLEMENTARY MATERIAL

See the supplementary material for additional figures that support the work presented within this article, including the aerosol droplet size distribution with images showing aerosolization patterns overtime, the overlaid immunofluorescent staining images supporting Figs. 5 and 7, the time at an air–liquid interface optimization data, a schematic and image of the airway barrier on a chip system, and the associated growth overtime data before challenge.

## Data Availability

The data that support the findings of this study is openly available in the University of Southampton repository at https://doi.org/10.5258/SOTON/D2252, Ref. 61.

## NOMENCLATURE

16HBE14o-: human bronchial epithelial cell line
ALI: air liquid interface
BCi: human airway basal cell line
FBS: foetal bovine serum
FNA: fuming nitric acid
HBSS: Hanks buffered saline solution
LLI: liquid liquid interface
MEM: minimal essential medium
NMP: N-methyl-2-pyrrolidone
OOC: organ on a chip
PBS: phosphate buffered saline
PCB: printed circuit board
PDMS: polydimethylsiloxane
PEEK: polyetheretherketone
Poly I:C: polyinosinic:polycytidylic acid
PVP: polyvinylpyrrliodone
SAW: surface acoustic wave
SPUDT: single phase unidirectional transducer
TER: trans epithelial electrical resistance
